# Supplementary material for: Are carbon emissions trading and green financial instruments synergistic? -Comprehensive quantitative research based on content analysis
Source: PLoS One. 2024 Mar 7;19(3):e0298601. doi: 10.1371/journal.pone.0298601 (PMC10919601; doi:10.1371/journal.pone.0298601)
Supplement: S1 Data — (ZIP) [file pone.0298601.s001.zip › Data/地级市Z_F工作报告.zip!/║■▒▒/╥╦▓2╩╨/└┤╫╘╣1⁄2╓┌║┼í╛┬φ┐╦╩2╛▌═°í┐_2014.docx]

各位代表：

　　现在，我代表市人民政府向大会报告工作，请予审议，并请各位政协委员和列席人员提出意见。

　　一、2013年工作回顾

　　2013年，市政府在市委正确领导下，在市人大和市政协监督支持下，紧紧团结依靠全市人民，按照“四个一流”的目标和要求，坚持竞进提质、效速兼取，沉着应对宏观环境复杂多变、经济下行压力加大的严峻挑战，统筹推进稳增长、调结构、促改革、惠民生，经济社会发展稳中有进、进中向好，主要经济指标增幅高于全国全省、领先沿江中部，实现现代化特大城市建设良好开局。完成生产总值2818亿元，同比增长11.5%；地方公共财政预算收入206.3亿元，增长34.6%；全社会固定资产投资2107亿元，增长30%；城镇居民人均可支配收入20934元，农村居民人均纯收入9121元，分别增长11.5%和13.4%；外贸出口20.2亿美元，增长21.2%；社会消费品零售总额860亿元，增长14.9%。市五届人大三次会议确定的目标任务基本完成。

　　(一)改革创新迈出新步伐。坚持解放思想，加强顶层设计，进一步完善现代化特大城市建设目标思路、政策框架、体制机制，宜昌新区、宜昌高新区、三峡枢纽港区、三峡旅游新区“四大平台”建设全面铺开。组建宜昌新区推进办公室、三峡枢纽港区管委会、三峡旅游新区管委会，建立高效决策机制。完善宜昌高新区管理体制，扩容白洋工业园、生物产业园，新建电子信息产业园。理顺市区两级事权和财权，调整完善城区财政体制，顺利完成城区建设管理体制、教育管理体制改革。编制千亿产业发展规划，出台扶持政策，精细化工、食品生物医药、先进装备制造、新材料、文化旅游、现代物流等六大千亿产业倍增工程全面推进。规范建设七大市级国有投融资公司，当年融资150亿元。创新重点项目推进体制机制，“重大项目建设年”活动扎实有效。加快食品药品监管体制改革，全国最佳食品安全放心城市建设深入推进。医药卫生体制、文化体制等各项改革进展顺利。加大直接融资力度，本土企业发行债券33.6亿元，13家企业在武汉股权托管交易中心挂牌。湖北华中文化产权交易所宜昌有限公司、武汉光谷联合产权交易所宜昌分公司挂牌成立。出台16条政策措施，支持科技创新。新认定国家高新技术企业63家，新增省级工程技术研究中心14家，新增孵化器、加速器面积25.5万平方米。新引进国家“千人计划”、省“百人计划”专家3人，新建院士专家工作站10家，新增省级以上专家17人。成功进入国家创新型试点城市和全国创建质量强市示范城市行列。

　　(二)发展质效实现新提升。工业加快转型升级。完成规模以上工业增加值1405.8亿元，增长13.7%。精细化工、先进装备制造、食品生物医药、新材料四大产业工业增加值占比超过70%。食品生物医药成为继精细化工之后第二个产值过千亿元的产业。我市入选全国战略性新兴产业集聚发展试点城市，成为全国唯一的新材料产业发展示范城市；被纳入全国老工业基地调整改造规划。预计实现高新技术产业增加值318亿元，占生产总值的比重提高到11.3%。宜昌高新区进入国家创新型特色园区行列。净增规模以上工业企业214家。产值过百亿元企业达到7家。3家企业入围中国民营企业500强。兴发集团获“长江质量奖提名奖”。中国驰名商标达到30件。

　　农村经济全面发展。粮食产量实现“十连增”，柑橘、茶叶、蔬菜、水产、畜牧产品产量持续增长，农业生产全面丰收。农产品加工产值达到1200亿元。新增省级以上农业产业化龙头企业16家。枝江酒业成为我市第二家年销售收入过百亿元农业产业化龙头企业。农民专业合作社达到4056家。家庭农场达到928家。农业机械化作业综合水平提高到61.5%。农户家庭承包耕地流转面积达到64万亩。申报实施土地整治56.8万亩。完成小型水库除险加固97座。新建、改造末级渠系1882公里。新解决13.2万人饮水安全问题。新增农村清洁能源入户工程3.7万户。

　　第三产业发展提速。第三产业投资占比达到47.3%，同比提高3.3个百分点。文化旅游业发展加快，产值达到416亿元，增长29%。全年接待游客3320万人次，旅游总收入260亿元，分别增长25.8%和29.8%。新增4A级景区3个、国家矿山地质公园1个，创建湖北旅游名村6家。产值过亿元文化企业达到16家。现代物流业完成产值717亿元，增长19.5%。云池港二期工程、三峡物流园农贸城建成运营，三峡翻坝物流产业园等园区建设加快。我市被列为国家二级物流园区布局城市。乡镇商贸中心和村级农家店覆盖率分别达到90.7%和83.6%。新建村级综合服务社217家。新增限额以上商贸流通企业740家。新增银行、保险、小额贷款公司等机构30家。金融机构存款余额2382亿元，贷款余额1749亿元，分别增长18.2%和19.8%。

　　项目建设成效明显。投资亿元以上在建项目900个，增长40.6%。成功举办京、港、粤、沪、汉等重大招商活动，引进项目687个，协议总投资4831亿元。招商引资到位资金930亿元。先后引进广汽轿车、南玻显示器件及光电玻璃、冠科电子等一批战略合作项目。南玻显示器件、三宁化工己内酰胺等项目竣工投产，人福药业出口药品基地、三峡云计算中心等项目加快建设。争取中央和省预算内投资项目1283个，无偿资金26.5亿元。争取三峡后续工作补助资金10.6亿元。落实三峡对口支援资金96.2亿元。全民创业全面推进，市场主体总数增长1.36倍，其中私营企业增长1.87倍。我市被评为2013年度中国最具投资吸引力城市。

　　县域经济快速发展。宜都、夷陵、当阳、枝江进入全省县域经济十强。17个镇进入全省镇域经济百强，数量居全省第一，其中陆城街道、樟村坪镇进入全省十强。宜都冲刺全国百强，其它县市赶超进位，经济社会发展均取得新成绩。

　　(三)城乡统筹取得新进展。宜昌城市总体规划和宜昌新区建设总体方案获省政府批准。首部城市色彩规划编制完成。城区实施城建项目183个，完成投资120亿元。坚持路网、公建、安置、生态“四个先行”，集中开工重大项目92个，新区建设初见成效。开工建设城市道路89.8公里。新区规划公建项目总建筑面积54.8万平方米。竣工和在建安置房3.8万套、面积383万平方米。扎实推进城市整理和绿化美化亮化工程，完成建筑综合整治122栋、亮化工程100处，建设景观式围墙1万米，定植绿化大苗2万株，初步建成沿江及城东大道等生态走廊。拆除违法建筑17万平方米、违法户外广告1万平方米。运河整治工程加快推进。城市综合管理考评连续三年位居全省前列，数字化城市管理信息系统建成并通过省级验收。

　　新型城镇化进程加快，宜都、枝江、当阳城镇建设实现新突破，五峰县城避险迁建进展顺利。重点中心镇、特色镇、宜居村庄建设加快，投入超过24亿元。龙泉、安福寺进入全省“四化同步”示范试点乡镇行列。新增省级生态乡镇2个、生态村6个。127个行政村被纳入全省“千村环境整治”试点。完成交通建设投资102亿元。宜巴高速公路基本建成，保宜高速公路路基全线贯通，宜张、宜岳高速公路建设提速。小鸦一级公路建成通车；318国道枝江段、当枝、三峡机场路等9条一级公路加快建设。庙嘴长江大桥建设进展顺利。生态文明建设扎实推进。宜昌中低品位磷矿综合利用入列全国首批示范基地。我市被确定为全国城镇低效用地再开发试点城市。夷陵、远安获全国国土资源节约集约模范县市称号。全市单位GDP能耗下降5%、地耗下降8%以上。植树造林24.77万亩。治理水土流失面积62平方公里。实施减排项目139个。城区PM2.5监测全面启动。节能减排年度任务全面完成。

　　(四)社会事业展现新进步。全年财政民生类支出247亿元，增长20.3%。城镇新增就业10.3万人，城镇登记失业率降低至3%。五项社会保险参保人数达到380.7万人次。城乡居民社会养老保险适龄人员参保续保缴费率达到99.9%。养老保险、医疗保险关系顺利实现转移接续。城乡居民大病保险制度全面建立，省内异地就医结算、全市医保“一卡通”基本实现。城镇居民、职工医保综合参保率达到98.9%。新型农村合作医疗参合率达到99.5%，政策范围内住院费用补偿比例达到75%。最低工资、社保待遇、城乡低保、医疗救助、临时救助等标准进一步提高。80岁以上高龄老人补贴制度实现全覆盖，惠及8.5万人。养老服务体系建设水平跻身全省前列。残疾人社会保障体系和服务体系基本建成。实施保障性安居工程2.6万套，其中实物建房竣工1.96万套，新增廉租住房租赁补贴2902户。新一轮棚户区改造正式启动。实施农村危房改造1.43万户。我市武陵山、秦巴山区域扶贫攻坚规划顺利实施。3.8万人口稳定脱贫。市场物价总体稳定。

　　学前教育加快发展，新建、改扩建公办幼儿园44所，学前三年毛入园率达到83%。9个县(市、区)通过国家义务教育均衡发展合格县市区复核认定。职教园建设加快，三峡旅游职业学院顺利入驻。三峡大学获得博士学位授予权。校舍安全工程基本完成。公共教育领域率先实行投资项目代建制。扶困助学工作实现全学段覆盖，资助学生31.9万人次、1.47亿元。县级公立医院综合改革试点顺利推进。实施基本公共卫生服务项目11类、国家重大公共卫生项目6项。在全省率先实现疾控“强基工程”先进县市达标全覆盖。公共文化服务体系不断完善。市博物馆新馆和奥体中心建设启动，市群艺馆新馆建成开馆，7个市县图书馆入选国家一级馆，广播电视无线覆盖工程全面启动，公共文化服务设施全部实现免费开放。成功举办第九届中国国际民间艺术节、第四届中国长江三峡国际旅游节、第三届宜昌长江钢琴音乐节、市第四届运动会等重大节庆活动。送戏下乡1806场。承办国家和省级体育赛事22项。《土家族撒叶儿嗬》获中国民间文艺最高奖“山花奖”，《五峰板凳龙》等3个节目获全国群星奖。群众文化体育活动蓬勃发展，全民阅读活动深入推进。新增省级非物质文化遗产名录7项、国家级文物保护单位5处。省社会科学普及教育基地在我市挂牌。

　　社会服务管理创新不断深化，城市网格化管理进一步完善，农村网格化管理全面启动，城乡网格员达到8709人。城市文明指数测评继续保持全省第一。罗长姐成为我市首位“全国道德模范”。城乡社区建设水平不断提高，秭归“幸福村落”创建经验走向全国。法治宜昌建设深入推进。安全生产形势总体稳定。率先在全省地市荣获全国社会管理综合治理最高奖项“长安杯”。

　　(五)行政效能得到新提高。再造行政审批流程，市级行政审批及管理服务事项办理时限压缩64%，政务服务标准化、信息化水平提升。建立诚信监管平台，行政审批前置中介服务进一步规范。清理规范行政权力，核减1710项，核减比例达到35%。党风廉政建设责任制全面落实，惩治和预防腐败体系建设、政风行风评议、行政监察和审计监督工作不断加强，治庸问责长效机制进一步健全。改进工作作风、密切联系群众的各项规定得到有效执行。认真听取人大代表、政协委员意见和建议，按期办复人大建议、意见和议案247件，政协建议案和提案475件，办结率100%。

　　服务三峡工程安全运行成效明显。援藏援疆工作深入开展。国防动员、民兵预备役建设和双拥工作不断加强。低生育水平持续稳定，生育文明建设和幸福家庭创建取得新突破。县级工商联建设经验在全国推广。对台、侨务、外事、保密、信访、统计、档案、方志、气象、人防、粮食、民族宗教、防震减灾等各项工作积极推进，妇女儿童、青少年、关心下一代等各项事业健康发展。

　　各位代表，这些成绩和进步，是在发展制约因素增多、阶段性特殊困难凸显的情况下取得的，来之十分不易，凝聚着全市人民的心血汗水，见证着方方面面的大力支持，展现出大城崛起的勃勃生机。在此，我代表市人民政府，向各位人大代表、政协委员，向广大干部群众、离退休老同志，向各民主党派、工商联、无党派人士、各人民团体，向驻宜解放军和武警官兵、中省驻宜单位，向所有关心、支持和参与宜昌建设的海内外朋友，表示衷心感谢并致以崇高敬意！

　　我们清醒地认识到，宏观环境依然错综复杂，区域竞争更加激烈，加快发展与转型发展的双重压力较大；产业整体竞争力还不够强，城乡二元结构矛盾依然突出，推进“四化同步”任重道远；资源约束强化，环境治理与生态保护任务十分繁重；财政实力、社保水平与民生需求尚有差距，提高居民收入和改善民生的任务仍然艰巨；政府职能转变有待进一步深入，发展环境有待进一步优化。对此，我们一定采取更加有力的措施，认真加以解决。

　　二、2014年工作重点

　　今年是深入贯彻落实党的十八届三中全会精神、全面开启改革新政的改革元年，也是现代化特大城市“三年出形象、五年成规模”的关键之年。中央决定在新的历史起点上全面深化改革，明确了改革的总目标、总任务，进一步释放改革动力、制度红利，开启了新一轮“改革窗口期”。国家依托长江黄金水道建设中国经济升级版支撑带，支持长江中游城市群建设，深入实施促进中部地区崛起战略，全面开展三峡后续工作；省委省政府贯彻落实习近平总书记视察湖北重要讲话精神，作出加快推进“建成支点、走在前列”的战略决定，加快实施“一主两副”、“两圈两带”区域发展战略。这些重大战略机遇正在宜昌形成叠加效应，必将为宜昌改革发展提供强大动力和广阔空间。近年来，我们不断创新发展思路，明确了“既大又强、特优特美”的现代化特大城市根本内涵，确立了“235”城市发展目标、“132”综合实力目标、“14567”具体工作构想，初步形成了“1＋10”改革总体部署，为今后一个时期的发展打下了战略性、开创性、长远性的良好基础。宜昌已经进入一个全新的发展阶段，我们对宜昌未来发展充满信心。

　　我们将以科学发展观为指导，全面贯彻党的十八大、十八届三中全会、省委十届四次全会和市委五届四次全会精神，深入解放思想，抢抓发展机遇，突出改革创新、转型升级、竞进发展，着力激发市场活力，着力统筹城乡发展，着力增进人民福祉，以更高标准、更大力度推进现代化特大城市建设，努力在全省“建成支点、走在前列”中率先突破，以宜昌之进、之转、之优服务全省发展大局。

　　今年，全市经济社会发展主要预期目标是：生产总值增长12%，全社会固定资产投资增长25%，社会消费品零售总额增长15%，地方公共财政预算收入增长12%，外贸出口增长12%，城镇居民人均可支配收入增长12%，农村居民人均纯收入增长12%以上，城镇登记失业率控制在4.2%以内，物价水平基本稳定，全面完成省政府下达的节能减排任务，保持经济持续健康发展、社会和谐稳定。

　　围绕实现上述目标，重点做好六个方面的工作。

　　(一)突出转型发展，加快提升产业实力

　　坚持把做大做强先进制造业、现代农业与发展现代服务业结合起来，大力实施高质量、可持续发展核心战略，加快提升传统产业、壮大千亿产业、培育新兴产业，全面提高产业发展质效。

　　进一步做强工业支撑。加强产业政策引导，推动主导产业延伸产业链、向价值链高端攀升。确保规模以上工业产值突破5000亿元，增加值增长15%。细化千亿产业推进措施，确保精细化工产业产值突破1500亿元，食品生物医药产业突破1350亿元，先进装备制造产业突破1000亿元，新材料产业突破600亿元。用足用好国家政策，重点支持发展石墨、磁电、氟基、硅基等特色新材料产业，加快培育一批产值过50亿元的龙头企业，建设具有国际竞争力的新材料产业聚集区。大力培育电子信息、新能源、节能环保等战略性新兴产业，尽快形成新的产业支柱。充分利用国家老工业基地调整改造政策，实施一批重大技术改造项目，推动一批企业“退城进园”、提档升级。积极化解过剩产能。继续实施优强企业倍增工程、中小企业成长工程，确保新增规模以上工业企业120家。广泛开展“质量兴企”活动，支持企业创建精品名牌，积极推广卓越绩效管理等现代管理方式，全面提高产品、工程、服务、环境质量，争创全国质量强市示范城市。加强生产要素协调，继续开展“进企业、送服务、解难题”活动，切实帮助企业解决实际困难和问题，保障企业正常生产经营。

　　加快推进农业现代化。围绕“农业强、农民富、农村美”目标，切实抓好“三农”工作。力争农业增加值增长5%，农村居民人均纯收入突破1万元。做大做强柑橘、茶叶、蔬菜、畜牧、水产、食用油六大百亿产业，加快培育花卉苗木、食用菌、魔芋、中药材四个新兴特色产业，加强农产品标准化基地建设。突破性发展农产品精深加工，深入实施百亿企业成长工程、农产品加工示范园区创建工程、名优品牌创建工程，壮大发展一批龙头企业，争创一批全省全国农产品加工示范园区，培育一批知名品牌。力争加工产值增长15%以上，新增中国驰名商标2件。全面深化农村改革，落实集体所有权、稳定农户承包权、放活土地经营权，鼓励农村土地承包经营权向专业大户、家庭农场、农民合作社、农业企业有序流转。加快建设完善市、县级农村产权综合交易中心。高标准改造基本农田40万亩。加强农田水利基础设施建设，除险加固中小型水库41座，新建、改造末级渠系600公里。解决10万人以上饮水安全问题。全面实施农村环境综合整治，开展畜禽养殖污染、白色污染及土壤污染治理。完成农村清洁能源入户工程2.5万户。力争新增省级生态乡镇2个、生态村6个、移民小康示范村5个。

　　大力发展现代服务业。抓住全省建设“一主两翼”长江中游商业功能区机遇，加快推进宜昌现代服务业园区等载体建设，加快培育科技金融、电子商务、信息技术和服务外包等现代服务业。全面推进三峡旅游新区建设，加快实施平湖半岛、游轮中心等重点项目，积极创建三峡国家级旅游度假区。启动全市旅游总体规划修编工作。深入开展A级景区、乡村旅游示范点、少数民族特色村寨建设，推动荷花镇、渔洋关镇、九畹溪镇等创建旅游名镇名村。确保全年接待游客3650万人次，旅游总收入300亿元。促进文化与旅游融合发展，支持国际生态智慧城、钢琴文化创意产业园、三峡国际珠宝博艺园、关公文化旅游城等项目加快建设，启动三峡广告创意产业园规划工作。组建宜昌三峡广电集团。稳步拓展文化产权交易所经营领域和范围。确保文化旅游业产值突破500亿元。抓住国家规划提高三峡枢纽货运通过能力的重大机遇，全面推进三峡枢纽港区建设，积极引进培育物流企业，大力发展临港、临空和临铁产业。确保现代物流业产值突破800亿元。积极推动总部经济、楼宇经济发展。加强企业上市培育工作，力争新增1家上市企业，2家企业进入上市辅导期，推动一批企业在“新三板”挂牌上市。加强金融信用体系建设，优化金融生态环境。加快现代流通体系建设，提高市场供应保障能力。加快发展电子商务，普及、深化电子商务应用。力争新增限额以上商贸企业200家。落实结构性减税政策，扩大营改增试点行业。

　　加强项目和园区建设。围绕“四大平台”建设和重点产业发展，精心策划一批重大项目，建立千亿产业项目库。抓好签约项目落地开工，加快推进冠科电子、广汽轿车、南玻平板显示等100个市级重点项目建设。优化项目建设环境，创新项目推进机制，对立项、用地、规划、施工等关键环节实行限时审批，切实提高服务效率。确保全年完成投资2600亿元以上。坚持产城共融，完善宜昌高新区园区发展规划，创新开发模式，拓宽融资渠道，加大园区基础设施、公共服务平台建设力度，增强配套服务功能。完善高新区经济社会管理、投融资体制，提高园区运转效率，激发园区科技创新活力。确保引进项目200个，协议投资500亿元。强力推进生物产业园集中连片开发。加快白洋工业园、电子信息产业园开发，尽快建成核心区。支持县市区建设专业化特色园区，引导企业和项目分类集中发展。推进五峰民族工业园、猇亭兴发工业园等园区建设，加快飞地经济发展。加快实施三峡机场改扩建项目，推动宜昌通航产业园建设。做强市级国有投融资公司，完成投融资150亿元。

　　(二)突出城乡一体，加快建设特大城市

　　坚持一手抓新区开发、一手抓旧城改造，一手抓中心城区建设、一手抓中小城镇发展，全力推进以人为核心的新型城镇化，加快宜昌现代化特大城市建设步伐。

　　强力推进宜昌新区建设。全面加快新区开发，为实现“三年出形象、五年成规模”奠定坚实基础。深化新区发展战略研究，贯彻后现代理念，走科学发展、绿色生态之路，打造生态型路网和节能型建筑，形成人与自然和谐发展的新区建设格局。注重城市个性和特色，依山就势打造宜昌建筑风格。进一步完善新区规划，加快伍家岗片区、点军片区等区域控制性详规修编，编制出台《宜昌新区建设标准》。实施96个重点建设项目，强力推进基础设施、大型公建、安置房、景观园林及综合体项目建设，确保全年完成投资264亿元。启动宜昌科技馆、大剧院、青少年教育实践活动基地等大型公建项目建设，加快建设奥体中心、博物馆、城市规划展览馆等大型公建项目，加快推进娃哈哈宜昌购物广场、中央商务区等社会投资项目。引进优质教育、医疗等机构进驻新区。坚持安置先行，全年续建、新建安置房700万平方米。

　　加快城乡基础设施建设。超前谋划，科学设计，着力构建功能完善的综合交通体系，打造国家综合运输通道关键节点。加快城区快速路网建设，打造峡州大道、江城大道、花溪路、西陵二路高架等“两纵两横”的城市环线。力争年内建成东山大道延伸段、柏临河路、东站路、共联路等东站片区道路，开工建设江南大道、双十路、南站路等点军片区道路。加快完善唐家湾片区和猇亭路网。着力建设东山大道快速公交系统。启动步行、骑行、景观绿道三位一体的绿道系统建设。新增公共停车泊位1000个。推动重点交通项目建设提速，确保保宜高速建成通车，加快建设宜岳高速、宜张高速、庙嘴长江大桥，开工建设香溪长江大桥，力争开工建设伍家岗长江大桥，加快推进白洋长江大桥、宜来高速、江北翻坝高速等项目前期工作。确保三峡机场路、陆渔路延伸段、白雅路建成通车。加快建设当枝一级路、318国道枝江段等一级公路，开工建设远安盘古至棚镇等一级公路。大力推进白洋、茅坪、红花套、峡口等10大港站建设。建设二级公路100公里、县乡公路80公里、通村水泥路600公里。推进城乡客运公交化改造，实施行政村“村村通客车”工程，实现现代运输网络城乡全覆盖。加快紫云铁路建设，积极争取和推进郑渝、宜常、翻坝等铁路项目。全面启动中心城区旧城改造工程，对旧城实施有机更新。推进环城南路、403厂等10大片区改造，完成开发改造100万平方米、整治维修120万平方米。深入推进城市整理和绿化美化亮化工程，完成建筑综合整治220栋，定植绿化大苗3万株，抓好重要节点夜景灯光建设。加快建设猇亭一水厂、点军供水供气等民生项目。实施农网升级改造工程，完成13个电网建设重点项目。加快建设以数字化、网络化、智能化为主要特征的智慧城市。

　　稳步推进新型城镇化。加快城乡规划、产业发展、基础设施和公共服务一体化进程，完善“一带四廊”、“一主五副”城镇体系，做强中心城区，做优县城，做特中心镇，做美中心村，提升城镇化发展质量，力争城镇化率达到58%。支持五峰完成县城避险迁建主体任务。扎实抓好龙泉、安福寺等11个新型城镇化试点。大力推进特色城镇和美丽乡村建设，继续实施村庄连片整治。加快发展县域经济，支持宜都争创全国百强县市，支持枝江、当阳、夷陵加快发展，支持远安、兴山、秭归建设山区生态经济强县，支持长阳、五峰建设武陵山科学发展示范区。扎实推进宜都、夷陵城乡统筹和点军都市农业等试验示范区建设。

　　(三)突出创新驱动，加快培育竞争优势

　　把创新驱动作为加快发展的总动力，摆在宜昌发展全局的核心位置，深化科技体制改革，促进科技与经济社会紧密结合，加快建设国家创新型城市。

　　着力提高自主创新能力。健全财政科技投入稳定增长机制，完善扶持政策，激发企业创新活力。深入实施科技型中小企业创新成长工程，大力发展高新技术产业，确保新增国家级高新技术企业40家以上，高新技术产业产值、增加值均增长20%以上，增加值占GDP的比重提高1个百分点以上。加强知识产权运用与保护，提升知识产权工作质量，积极创建国家知识产权示范市。围绕重点产业、新兴产业，健全产学研协同创新机制，实施一批重大科技创新项目，突破一批核心关键技术，促进科技创新成果在宜就地转化。

　　加大创新载体建设力度。突出高端产业发展和区域创新功能，推进科技项目向“四大平台”聚集。启动宜昌科教城建设。鼓励县市区工业园区建立健全研发、检测、孵化等创新体系，建设创新型园区。支持企业技术创新平台建设，新增省级以上工程技术研究中心6家以上，新建市级工程技术研究中心、重点实验室8家以上。推进院士专家工作站和博士后科研工作站建设。引进培育高端研发、科技孵化等创新载体，确保新增孵化器、加速器面积20万平方米，在孵企业总数300家以上。

　　推动科技金融深度融合。推动科技资源与金融资源有效对接，加快建设多元化、多层次、多渠道的科技投融资体系。积极引进服务科技型企业的各类专业金融机构，支持有条件的银行、保险公司、担保公司设立科技金融专营机构。大力创新金融服务，综合采用买卖方信贷、知识产权和股权质押贷款、企业债券、集合信托等方式，支持科技型企业开展技术创新融资。发挥市级国有投融资公司作用，采取阶段参股等方式，支持科技型中小微企业发展。鼓励风险投资机构、民间资本通过股权投资等形式参与孵化器建设，增强对在孵企业的投融资服务。

　　优化人才创新创业环境。大力实施“宜昌英才工程”，提升品牌影响力，深入推进人才强市改革试验区建设。健全支持人才创新创业创优的政策机制，大力引进以产业领军人才和高层次创新创业人才为重点的各类人才，大幅提升人才总量和质量。支持高层次人才创办科技型企业。实施产业创新团队培育、紧缺专业技术人才培养等重大人才工程。加快人才公寓示范小区、青年人才公寓建设，着力改善人才工作生活条件。创新人才工作体制机制，提升服务人才工作水平。倡导崇尚成功、宽容失败的理念，在全社会营造宽松自由的创新创业氛围。

　　(四)突出改革开放，加快释放发展活力

　　坚持把改革贯穿于经济社会发展各个领域各个环节，扩大对内对外开放，增强社会活力，增强发展动力，为建设现代化特大城市拓展更加广阔的空间。

　　全面推进重点领域改革。按照市委“1＋10”的改革总体部署，突出问题导向，全面深化改革，创新体制机制，再造现代化特大城市建设新优势。围绕推进以人为核心的新型城镇化，创新户籍管理、社会保障、土地管理等制度，促进城乡一体化发展。以转变政府职能为突破口，大力简政放权，深化行政审批制度改革，加强事中事后监管，提升行政效能，着力打造“低成本、高效率、国际化、法治化”的一流发展环境。深入推进政府机构改革，优化机构设置，严控人员编制，分类推进事业单位改革。优化城区管理体制，下放部分市级职责到区，强化区级政府职能职责，建立事权和支出责任相适应的制度。推进食品药品监管体制改革，实施食品安全网格化管理、溯源管理和诚信管理，构建现代食品安全治理体系。建立和完善最严格的源头保护制度、环境治理和生态修复制度、损害赔偿与责任追究制度，用制度保护生态环境。完善财政预算绩效管理，建立政府重点投资项目财政监管机制。推行市级财政部分专项资金竞争性分配改革。改革商事登记制度，降低市场准入门槛，促进市场主体增量提质。进一步畅通民间资本投资渠道，激发投资活力，鼓励、支持、引导非公有制经济发展。深化国有企业改革，完善现代企业制度，增强国有经济活力和创造力。统筹推进其他各项改革。

　　全面提升招商引资质效。坚持权利与责任对等、招商信息与资源共享，统筹推进市县、市区联动招商。创新招商项目评审、推进、问责机制，注重聚商选资，提高招商实效。力争全年新开工亿元以上招商项目360个，招商引资实际到位资金1080亿元。争取三峡对口支援到位资金70亿元。围绕六大千亿产业发展，大力引进产业链和关联企业投资项目。充分发挥在宜行业商会、协会和企业家的作用，推进以商招商、委托招商。加强与武汉东湖高新区、上海临港工业园合作，力争引进一批高新技术项目和产业转移项目。加强与三峡集团、葛洲坝公司等中省企业战略合作，力争新增合作投资60亿元以上。

　　全面提高对外开放水平。坚持对内对外开放并举，放宽开放领域，提升开放层次，以开放促改革、促发展，大力培育参与国际经济合作竞争的新优势，提高宜昌国际化水平。增强宜昌在鄂西地区城市融合发展中的牵引作用，努力建设长江中游城市群的西部支撑点。扩大与长三角、成渝城市群合作领域，加强与长江经济带各城市的有效对接，积极参与区域产业分工合作与交流，打造内陆沿江开放高地。加强出口基地建设，搭建公共服务平台，提高出口产品竞争优势和效益。主动对接上海自由贸易试验区，加快推进宜昌综合保税区申报工作，强化口岸建设，促进便捷通关。努力创造条件增加国际客货运航线。支持企业开拓境外市场。扩大旅游、文化、工程、劳务等服务产品出口，增加服务贸易份额。

　　(五)突出绿色发展，加快打造美丽宜昌

　　把生态文明建设放在更加突出位置，强化环境治理与生态保护，确保通过国家环保模范城市复核检查，积极创建国家生态园林城市，进一步彰显生态宜居城市魅力。

　　加强生态建设和环境保护。强化山体水体保护利用，开工建设求雨台、城东、柏临河、卷桥河等公园项目，规划建设胭脂坝湿地公园，推进火车东站片区水景观建设，抓好主要道路彩色森林景观建设。全面推进点军绿色低碳生态新区建设。稳步实施退耕还林、天然林保护、长江防护林建设等重点生态工程，完成植树造林20万亩，切实加强森林资源管理。完成治理水土流失面积50平方公里。严格执行主体功能区规划。完成城市环境总体规划编制。加强三峡库区、清江库区、长江沿岸等生态敏感区的保护修复。实施长江岸线治理工程。抓好域内重点流域综合治理。完成运河综合整治。强化重点集中式饮用水源地保护。加强地质灾害防治。深入推进大气污染联防联控，启动机动车环保标志管理，加强建筑施工扬尘监控和工业废气治理。定期发布环境质量监测数据。加强环境卫生监测和管理。严肃查处环境违法突出问题。

　　推进节能减排和资源利用。加快淘汰落后产能。加强对重点污染排放企业跟踪监管。加强重点用能企业能源在线监测，抓好公共机构节能工作。大力实施绿色节能建筑建设工程，新增可再生能源建筑应用面积146万平方米，推进建筑节能改造18万平方米，发展绿色建筑9万平方米。大力发展循环经济，抓好猇亭园区循环化改造试点，加快建设一批生态工业园区和再生资源基地。抓紧建设磷矿资源交易平台。实施最严格的耕地保护和水资源管理制度。继续开展城乡建设用地增减挂钩、低丘缓坡地综合开发利用、城镇低效用地再开发三项创新试点，推进旧城镇、旧厂房、旧村庄改造，确保全市单位GDP地耗下降8%以上。

　　抓好城市管理和环境整治。完善城市管理工作考评制度，健全城市管理长效机制。提高数字城管运用水平。开展违法建设治理、市容环境整治、交通秩序规范、文明素质提升等城市环境综合治理行动。加快完善城市污水管网，提高污水处置收集率。开展城区主排水管网清疏，增强城区排水防涝能力。完善生活垃圾收运处置体系，建成3座城区垃圾转运站，开工建设黄家湾垃圾填埋场一期封场项目，加快实施猇亭垃圾处理场项目，确保城区生活垃圾无害化处理率达到95%。抓好全国餐厨废弃物资源化利用试点和全省生活垃圾分类试点工作。提高环卫作业机械化水平，确保道路机扫率达到55%。

　　(六)突出民生改善，加快增进人民福祉

　　坚持尽力而为、量力而行，把有限的财力更多地用到改善民生上，努力解决人民群众最关心最直接最现实的利益问题，让改革发展成果更多更公平地惠及全市人民。

　　促进城乡充分就业。建设城乡一体的人力资源市场，继续开展城乡劳动者职业技能培训，强化公共就业服务和企业用工服务，统筹做好高校毕业生、城镇适龄青年、农村转移劳动力、城镇困难人员等群体就业工作，加强退役军人、随军家属就业安置，推动实现更高质量的就业。扶持小微企业发展，鼓励“二次创业”，大力促进创业带动就业，积极创建国家级创业型城市。确保新增城镇就业7.7万人。健全进城务工人员工资支付保障机制和职工工资正常增长机制，巩固和谐劳动关系。多渠道增加居民财产性收入。扎实推进新一轮扶贫开发，实施30个特困村脱贫攻坚行动和86个村扶贫开发整村推进，稳定脱贫3.5万人。扎实做好移民后期扶持工作，促进移民安稳致富。

　　完善社会保障体系。不断扩大社会保险覆盖面。城乡居民社会养老保险适龄人员参保续保率保持90%以上。积极实施新型农村合作医疗市级统筹。全面实施大病医疗保险制度。建立以公共租赁住房为主体的住房保障分配体系，探索共有产权保障模式，完善产权分配和上市交易调节机制，逐步解决中低收入家庭的住房困难。申报建设各类保障性住房6.4万套。改造农村危房1.5万户。加快完善社会和居家养老服务体系，城市社区养老服务中心、农村老年人生活互助照料中心覆盖率分别达到80%和40%。确保社会救助、优抚安置、社会福利等保障标准与城乡居民平均生活水平同步提高。

　　大力推进文化建设。培育和践行社会主义核心价值观，推动学习道德模范、学雷锋志愿服务活动常态化，不断提高市民文明素养。扎实做好全国文明城市复查迎检工作和未成年人思想道德建设测评工作。全面启动国家公共文化服务体系示范区创建三年行动计划。加快推进市音乐厅、广播电视发射塔、新闻中心、体育场改造等公共文化体育设施建设。实施数字文化建设计划。建设一批特色文化社区、特色文化村。扶持民办文化事业发展。办好宜昌市首届艺术节、屈原故里端午文化节、长江钢琴音乐节等重大节庆活动，深入开展“文化宜昌·全民阅读”活动。积极备战省十四届运动会，促进竞技体育、群众体育和体育产业协调发展。加强文化遗产保护、传承与利用。强化文化、新闻出版市场监管，培育健康向上的网络文化。繁荣发展哲学社会科学、文学艺术事业。

　　全面发展社会事业。落实教育优先发展战略，加大教育投入力度，加快推进教育现代化，着力打造宜学之城。成立宜昌市英才教育基金会。实施第二期学前教育三年行动计划，扩大公益性、普惠性幼儿园覆盖面。推进义务教育均衡发展，加大现代化学校创建力度。加快推进市一中、市十三中学整体迁建，完成职教园一期工程。加快高等教育发展，支持三峡大学办出特色争创一流。推进高等教育、职业教育产教融合，服务地方经济发展。深化医药卫生体制改革，着力推进县级公立医院综合改革。优化城区医疗卫生资源布局，加快市中心医院及点军分院、市一医院等医疗机构基础设施建设，启动市妇女儿童医院建设，建成市残疾人康复医院，鼓励兴办民营医疗机构，努力打造区域性医疗卫生中心。全面提升基层医疗卫生服务水平，完善城区社区卫生服务体系，加强村卫生室和乡村医生队伍建设，为城乡居民提供安全有效、方便价廉的基本医疗卫生服务。巩固提升国家卫生城市创建水平，努力争创全省健康城市。坚持计划生育基本国策，创新规范计划生育社会化服务管理体制机制，促进人口长期均衡发展。扎实做好民族团结进步和援藏援疆工作。依法管理宗教事务。进一步抓好统计、档案、方志、科普、慈善、外事、侨务、港澳、对台、保密、消防、气象等各项工作。深化全民国防教育，加强国防动员、民兵预备役和人民防空工作，支持驻宜部队现代化建设，巩固发展军政军民团结，推动军民融合深度发展。

　　改进社会治理方式。坚持系统治理、依法治理、综合治理、源头治理，巩固“一本三化”服务体系，推进网格化管理向葛洲坝区域和全市农村全覆盖，努力把宜昌打造成全国社会建设的典范。创新有效预防和化解社会矛盾体制，强化重大决策社会稳定风险评估，实行网上受理信访制度，建立畅通有序的诉求表达、矛盾调处机制，使群众问题能反映、矛盾能化解、权益有保障。完善立体化社会治安防控体系，依法防范和惩治违法犯罪活动，推进法治宜昌建设，巩固“长安杯”创建成果，加快把宜昌打造成“平安中国”建设示范区。完善社区矫正制度。加强和改进流动人口、特殊人群服务和管理。维护三峡库坝区社会稳定，服务三峡工程安全运行。积极发挥工会、共青团、妇联、工商联等人民团体和社会组织在社会治理中的重要作用。强化城乡社区自治服务功能，提高社区服务水平。加强社会诚信体系建设。深入开展食品药品安全专项整治，进一步加大对违法行为惩处力度，推进示范创建，打造一批食品药品放心品牌，加快建设全国最佳食品安全放心城市。加强应急管理和防灾减灾体系建设。强化安全生产红线意识、责任意识、防范意识，着力推进重点行业、重点领域安全生产专项整治，加快隐患排查治理标准化、数字化体系建设，健全安全生产长效机制，坚决杜绝重特大事故发生。

　　三、加强政府自身建设

　　扎实推进党的群众路线教育实践活动，进一步转变政府职能，创新行政管理方式，提高科学管理水平，更好地发挥政府作用，努力建设法治政府和服务型政府。

　　(一)切实做到勤政为民。认真落实中央、省委、市委关于改进工作作风、密切联系群众的各项规定，带着感情下基层，转变作风办实事，争做信念坚定、为民服务、勤政务实、敢于担当、清正廉洁的好干部。全面完成市、县、乡、村四级政务服务中心标准化、信息化建设，加强电子政务建设，采取线上线下多种服务方式，向社会提供全方位、高质量政务服务。科学配置各级平台政务服务事项，将涉及民生的公共服务事项尽量下放，集中进入街道、社区和乡村政务服务中心办理，方便群众就近办事。规范有序开展政府向社会力量购买公共服务。健全抓落实责任体系，打造落实链条，以踏石留印、抓铁有痕的工作力度抓好各项工作落实。扎实开展治庸问责，着力治理庸懒散奢等不良风气，着力提高行政机关服务效能。严格行政问责追责，确保令行禁止、政令畅通。

　　(二)全面推进依法行政。认真开展法治政府建设试点工作。把依法行政贯穿于政府决策、执行、监督全过程和政府管理各环节，严格按法定权限、程序办事履职。认真执行市人大及其常委会的各项决定和决议，主动听取人民政协的意见和建议，切实办理好人大建议、意见、议案和政协建议案、提案。支持审判机关和检察机关依法独立行使审判权和检察权。加强和改进政府法制工作，做好较大的市申报工作。减少行政执法层级，加强重点领域基层执法力量。规范执法自由裁量权，推进行政执法、处罚结果公开。健全行政执法责任制和执法过错追究制。健全科学民主决策机制，完善行政决策程序。

　　(三)始终保持清正廉洁。推行政府权力清单制度，依法公开权力运行流程。完善政务和各领域办事公开制度，推进决策公开、管理公开、服务公开和结果公开，让人民监督权力，让权力在阳光下运行。加强电子监察系统建设，实行行政审批全过程在线监察。强化效能监察、审计监督，推进工程建设、土地、产权、政府采购等公共资源交易规范管理。全面落实《党政机关厉行节约反对浪费条例》，健全严格的财务预算、核准和审计制度，着力控制“三公”经费和一般性支出。严格执行党风廉政建设责任制，加强干部队伍纯洁性建设。严肃查处各类违法违纪案件，着力解决损害群众利益的突出问题。

　　各位代表！时代赋予重任，奋斗铸就辉煌。让我们把思想和行动统一到中央、省委和市委关于全面深化改革的重大决策部署上来，进一步强化进取意识、机遇意识、责任意识，解放思想，与时俱进，众志成城，奋发图强，为加快建设“既大又强、特优特美”的现代化特大城市而努力奋斗！
